# Molecular mechanisms of immunotherapy resistance in triple-negative breast cancer

**DOI:** 10.3389/fimmu.2023.1153990

**Published:** 2023-06-23

**Authors:** Yiwen Zheng, Shujin Li, Hongchao Tang, Xuli Meng, Qinghui Zheng

**Affiliations:** ^1^ The Second Clinical Medical College, Zhejiang Chinese Medical University, Hangzhou, China; ^2^ General Surgery, Cancer Center, Department of Breast Surgery, Zhejiang Provincial People's Hospital (Affiliated People's Hospital, Hangzhou Medical College), Hangzhou, Zhejiang, China

**Keywords:** immunotherapy, triple-negative breast cancer, resistance mechanism, tumor microenvironment, antigen presentation

## Abstract

The emergence of immunotherapy has profoundly changed the treatment model for triple-negative breast cancer (TNBC). But the heterogeneity of this disease resulted in significant differences in immunotherapy efficacy, and only some patients are able to benefit from this therapeutic modality. With the recent explosion in studies on the mechanism of cancer immunotherapy drug resistance, this article will focus on the processes of the immune response; summarize the immune evasion mechanisms in TNBC into three categories: loss of tumor-specific antigen, antigen presentation deficiency, and failure to initiate an immune response; together with the aberrant activation of a series of immune-critical signaling pathways, we will discuss how these activities jointly shape the immunosuppressive landscape within the tumor microenvironment. This review will attempt to elucidate the molecular mechanism of drug resistance in TNBC, identify potential targets that may assist in reversing drug resistance, and lay a foundation for research on identifying biomarkers for predicting immune efficacy and selection of breast cancer populations that may benefit from immunotherapy.

## 1 Introduction

### 1.1 Triple-negative breast cancer

Breast cancer is the most common cancer among women worldwide and the second most common cause of cancer death (1). There are several subtypes of breast cancer, including luminal A/B, HER2-positive, and triple-negative breast cancer (TNBC), depending on the expression of estrogen and progesterone receptors (ER, PR) and the human epidermal growth factor receptor-2 (HER2). Breast cancer is a highly heterogeneous disease with large differences in molecular characteristics and prognosis between the different subtypes (2). Triple-negative breast cancer, as its name indicates, is defined as breast cancers that are negative for all three proteins, ER, PR, and HER2 (by immunohistochemistry or FISH), and accounts for approximately 15% of breast cancer patients worldwide. Compared with other subtypes, TNBC phenotypes are basal-like with high proliferative activity, high infiltration of immune cells, and homologous recombination defects associated with BRAC mutation (2). The metastatic and recurrence rates are high and patients typically have lower survival rates (3). The standard treatments for breast cancer include surgery, chemotherapy, hormone and targeted therapy. However, the absence of hormone receptors and HER2 significantly reduces the number of effective therapeutic options for TNBC, and surgery and chemotherapy are still the main treatment methods (4, 5).

### 1.2 Immunotherapy of TNBC and its dilemma

TNBC is more immunogenic than the other breast cancer subtypes (6) owing to the production of more neoantigens as a result of the high mutational burden and genome instability in this tumor type. Thus, the tumor microenvironment (TME) of TNBC is usually enriched in tumor-infiltrating lymphocytes (TILs) (7), making them particularly attractive for early immunotherapy clinical trials (8–10). Furthermore, PD-L1 is commonly overexpressed in TNBC compared to other subtypes and is significantly associated with the presence of TILs.

In view of these immunogenic features of TNBC, immune checkpoint blockers (ICBs) targeting PD-1/PD-L1 etc. have shown great promise in the treatment of breast cancer. This overturn past views of breast cancer as immunologically “cold”, a generalization that ignores the subtype heterogeneity of breast cancer and the complexity of patients’ immune status. Although some immune activity was observed in advanced metastatic TNBC, there are still limitations in the treatment response rate. A multicenter, non-randomized Phase Ib trial (KEYNOTE-012) showed an overall response rate of less than 20% in advanced triple-negative breast cancer expressing PD-L1 (10). Another reasonable treatment strategy is adoptive T cell therapy (ACT). However, TME within breast tumors are particularly adverse to reintroduced “enhancer” effector cells. The synthesis of immunomodulatory components (e.g. inhibitory cytokines, biochemical reaction products, etc.), the expression of immune checkpoint molecules, and the direct cytotoxic activity of TME with respect to effector cells create barriers to adoptive therapeutic agents (11). Therefore, the basic mechanisms controlling immune resistance to breast cancer still need to be answered to help expand the efficacy of immunotherapy in patients with triple-negative breast cancer.

### 1.3 Tumor immunoreactivity: “hot” or “cold” tumors

Although the emergence of immunotherapy has provided new options and hope for patients with different types of cancers, only a small percentage of cancer patients are currently benefiting from this (12). Many studies have confirmed that the abundance of TILs, especially T cells, in the TME can predict good response for immunotherapies. These tumors are considered as immunoreactive or “hot” tumors, while those that lack such TILs are termed “cold” tumors (13). Based on this concept, expression of CD3 and CD8 positive T cells in and around the tumor has been developed into an “immunoscore” to classify tumors (14, 15). However, immune reactivity is affected by various factors such as the host, tumor, and internal environment. Therefore, it is necessary to comprehensively consider these immune characteristics to determine whether a tumor will be responsive to immunotherapy. The combination of these factors represents the immune status of the tumor. That is, the balance between tumor-promoting and tumor-suppressing immune factors.

## 2 Molecular mechanism of drug resistance in TNBC

### 2.1 External mechanism of TNBC immune resistance

The immune system is thought to be able to accurately identify and eliminate tumor cells that are different from healthy cells under normal circumstances, and development of cancer arises from disruptions of the tumor surveillance function of the immune system. Some tumors can harbor a high percentage of tumor-infiltrating lymphocytes (TILs), which have been shown to be significantly correlated with the prognosis of breast cancer and other malignancies. Many types of lymphocytes can infiltrate the tumor, including effector T cells (Teffs), regulatory T cells (Tregs), B cells, natural killer cells (NKs), antigen-presenting cells (APCs), macrophages, etc. and they may have both pro- and antitumor effects. T cell-mediated adaptive immunity and NK cell-mediated innate immunity play important roles in the fight against tumors.

#### 2.1.1 T cell-dominated adaptive immune resistance

T cells are the predominant cytotoxic lymphocytes of the immune system, and activation of the immune system by tumor cells mainly involve this cell type. Tumor-specific antigens (TSA) are normally presented by APCs, leading to the activation and recruitment of T cells, which recognize and eliminate the cancer cells (16). The abundance of T cells have been shown to predict the overall treatment response and the overall survival rate of breast cancer patients. Monitoring of immunotherapy responsiveness also mainly revolves around T cell abundance (17), and may include immunophenotyping of these infiltrated T lymphocytes in colorectal cancer (18). However, due to the heterogeneity of the TIL composition and the diversity of T cell phenotypes, it is far from sufficient to use T cell abundance alone to represent the immune state of the tumor. Typically, both stimulatory and suppressive immune cells are present in tissues and organs, and a balance of their functions maintains the normal human environment. Similarly, stimulatory and suppressing T cells are present in tumors, and higher ratios of immunostimulatory subsets (Th1) to immunosuppressive ones (Treg and Th2), to some extent, indicates antitumor activities and better prognosis (19). However, not all T cells within the tumor are active, i.e. able to recognize tumor antigens; the role of these “bystander” T cells in normal immune responses, antitumor activities and immunotherapy is unclear (20) and more research is required. Exhaustion is another characteristic of tumor-infiltrated T cells and impairments in the memory subset and reduced lifespan of proliferative T cells are often observed in therapeutic resistant patients (21, 22). Thus, the goal of ICB treatment is to restore the function of these exhausted T cells, and its effectiveness relies heavily on the ability of T cell to expand, activate and form memory cells; damage to any of these mechanisms can lead to resistance to immunosuppressive therapies in TNBC (23).

At present, programmed cell death protein 1 (PD-1) and its programmed death-ligand 1 (PD-L1) expressed on the surface of activated T cells are the main ways to regulate the activity of T cells. Under normal physiological conditions, PD1/PD-L1 inhibitory co-stimulatory signals prevent T cells from becoming uncontrollably over-activated to attack normal cells. However, the overexpression of PD1/PD-L1 on the surface of tumor cells can induce T cell exhaustion and enable tumor cells to escape T cell immune attack. Therefore, PD1/PD-L1 pathway blockers, including PD-1 monoclonal antibody and PD-L1 monoclonal antibody, have become important targeted drugs for breast cancer immunotherapy. These drugs restore anti-tumor immunity by blocking PD1/PD-L1 signaling axis to reactivate exhausted T cells in the tumor immune microenvironment. PD-L1 has become an important molecule in tumor immunology research. Understanding the mechanism of PD-L1 expression regulation is of great significance for improving the efficacy of PD1/PD-L1 targeted therapy and avoiding immune escape of tumor cells. The regulation of PD-L1 expression can occur in five links: chromatin changes, genome changes, transcription factors and post-transcriptional regulation, translation and post-translational regulation, and induction of tumor microenvironment. First, the CD274 gene encoding PD-L1 is located on chromosome 9p24.1, and changes in chromatin structure and properties in this region directly affect gene expression, including chromatin modification and rearrangement. Second, the abnormal expression of PD-L1 is often caused by changes in any step of the genome transcription and translation process, in which the abnormal activation or inactivation of the signaling pathway can affect the activity of the body’s immune function. Third, the excessive secretion of INF-γ, TNF-α, interleukin and other pro-inflammatory cytokines in tumor microenvironment can induce the expression of PD-L1 in tumor cells through different signaling pathways and promote immune escape (24, 25).

In conclusion, TILs are heterogeneous in their phenotype and function, and a delicate balance between immunostimulatory and immunosuppressive T cells determines the overall immunogenic status of the TME (26). Therefore, assessment of the tumor microenvironment should consider the abundance, subsets and their proportions, and the distributions of TILs comprehensively to identify truly immunogenic TNBC, which may help to accurately identify TNBC patients who will benefit from immunotherapy and improve their outcomes.

#### 2.1.2 NK cell-dominated innate immune resistance

The innate immune system, including NK cells, APCs, macrophages, and neutrophils, have been shown to exert antitumor effects independently of adaptive immunity (27). As the only member of the innate immune system with cytotoxic effects, high infiltration of NK cells is often associated with good prognosis in TNBC (28). However, NK cells are generally rare among breast cancer TILs, accounting for only around 5% (29); the TNBC subtype tends to be more significantly associated with higher NK cell infiltration (30).

Although they can function independently, innate and adaptive immunity are by no means isolated from each other, and there are complex interaction networks between them. Since NK cells lack T cell receptors (TCRs), they do not recognize tumor cells *via* the major histocompatibility complex class I (MHC-I) molecule, and can instead target malignant cells that have shed MHC (31, 32). This direct cytotoxic mechanism complements adaptive immunity and opens up alternative therapeutic avenues for cancer patients who are resistant to ICB therapies through adaptive mutations. In addition to direct cytotoxicity, NK cells can also regulate cytokine and chemokine secretion. For example, secretion of interferon gamma (IFN-γ) promotes the maturation of dendritic cells (DCs), stimulates helper T cell function (33), and increases the expression of MHC-I on tumor cells, thereby increasing their susceptibility to T cells (34, 35). However, NK cells can also block T cell activation by enhancing the expression of PD-L1 and LAG-3, and stimulating angiogenesis, thereby promoting immune escape. Notably, tumors with high NK infiltration are associated with low T cell infiltration (36).

NK cell recognition of tumor cells relies on a set of inhibitory and stimulatory receptors that monitor the expression of ligands associated with oncogenic transformation of proximal cells. Reduced expression or deletion of NKG2D, the main activating receptor for NK cells, can prevent NK cells from exerting its innate immune functions, while targeting the proteolysis site of MICAα3 to block the shedding of the NKG2D ligands, MICA and MICB, can improve the antitumor activity of NK cells in mice (37). In short, the innate antitumor immunity of NK cells is worthy of recognition, but the role and mechanism of NK cells in mediating immune resistance are still not very clear. Development of immunotherapy targeting NK cells will help to overcome some deficiencies of classical immune checkpoint blockade therapies, improve the immune resistance of TNBC, and improve treatment options for breast cancer patients.

#### 2.1.3 Immunomodulatory roles played by other cells

Accumulating evidence indicates that complex cell populations in the TME are involved in immune activities. Although T cells are central to antitumor immunity, other cell types present in the TME also play immunomodulatory roles; tumor-associated macrophages (TAMs) and myeloid-derived suppressor cells (MDSCs), etc., have been shown to have immunosuppressive properties.

Tumor associated macrophages are very abundant in tumor and are the main components of inflammatory cells. The understanding of TAM’s function was initially limited to its antitumor effect. But as the research progressed, it has been found that macrophages can be polarized to different subtypes under the action of various microenvironmental stimuli. The M1 subtype are generally pro-inflammatory, while the M2 subtype are anti-inflammatory. TAMs stimulated by the hypermutated environment of the tumor are often polarized to the M2 subtype (38) and participate in tumor immune escape by producing cytokines such as IL-10, PGE2, and TGFβ, and are closely related to poor prognosis of many cancers and the occurrence of drug resistance (39). A recent study demonstrated a temporal and spatial correlation between TAMs and CD8+ T cell depletion in cancer. This study reveals a mechanistic link to a positive feedback loop driven by antigen-specific synapses, and provides a possible pathway by which TAMs, in conjunction with the oxygen-poor environment within tumors, promote depletion of CD8+ T cells, thereby promoting initial and sustained tumor immune escape (40).

Myeloid-derived suppressor cells originated from pathologically activated neutrophils and monocytes and were identified and named for their strong immunosuppressive activity. MDSC interferes with the activation of immune functions through crosstalk with other immune cells. They can increase PD-L1 expression on T cells to induce anergy (41), recruit other immunosuppressive cells, such as Tregs and TAMs, and stimulate the proliferation of these cells to promote immunosuppression (42, 43).

**Figure 1:**
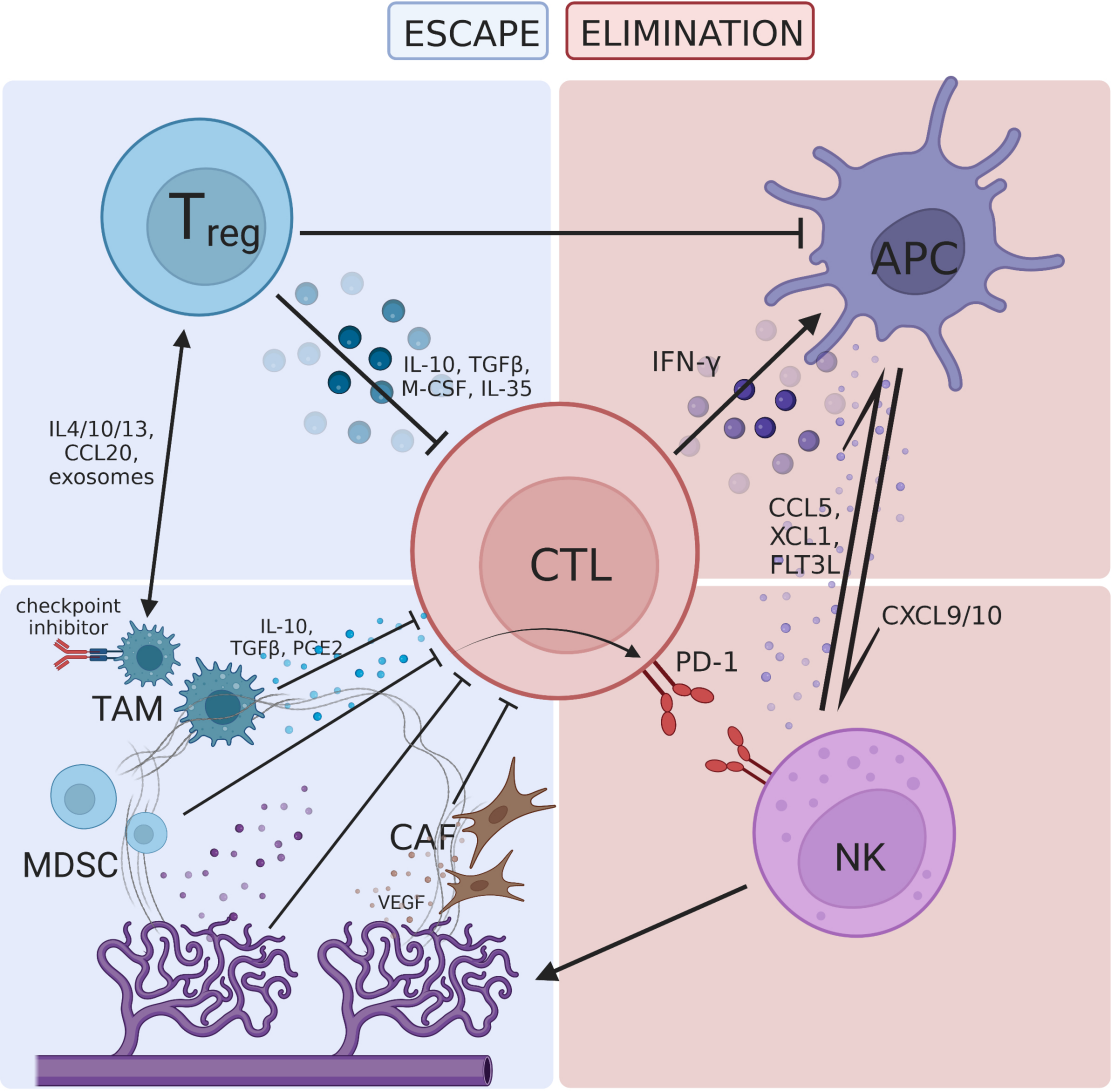
The tumor microenvironment is regulated by various immune-promoting and immune-resistant components, forming a complex interactive network. **(A)** The immune-promoting components include cytotoxic T lymphocyte (CTL), antigen presenting cell (APC) and natural killer cell (NK). CTL plays a central role in immunotherapy and interacts with APC and NK through cytokines. **(B)** Immunoregulatory cells include regulatory T cells (Treg), tumor-associated macrophages (TAM), and myeloid-derived suppressor cells (MDSC), which inhibit T cell proliferation through direct pathways such as expression of immune checkpoint molecules and immunosuppressive cytokines. Dysfunctions in these cells can also create an immunosuppressive TME through indirect pathways such as crosstalk with Treg and hijacking of the PD-1 pathway; **(C)** cancer-associated fibroblast (CAF) and abnormal tumor neovascularization hinder T cell infiltration. Hypoxia induced metabolic defects increase the acidity of the TME, forming a barrier for antitumor immune activities.

In summary, immunoregulatory cell subsets in the TME jointly construct an immunosuppressive network that weakens the antitumor effect of the host immune system (44) **(** **Figure 1** **)**.

### 2.2 Intrinsic factors of TNBC immune resistance

#### 2.2.1 Loss of tumor antigen presentation

**Figure 2:**
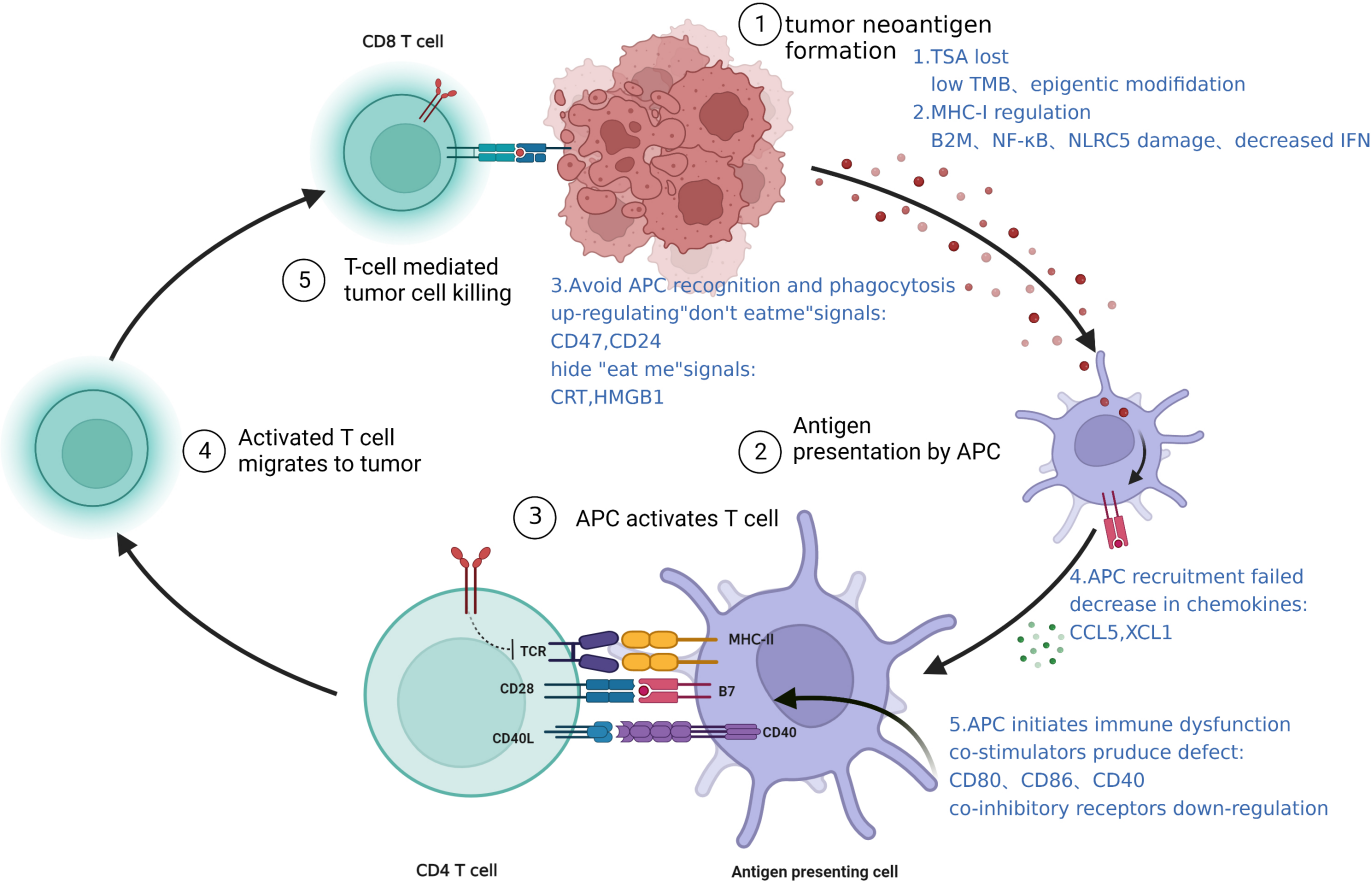
Intrinsic mechanisms of immune resistance: a schematic representation of antigen presentation defects.

Antigen presentation is the first step in the activation of the immune system, and involves APC recognition of tumor antigens; recruitment, activation, and maturation of APCs; APC presentation of antigens to T cells; cross-presentation of antigens. Failure of any one step in this process can lead to immune evasion **(** **Figure 2** **)**.

**The alteration of antigen MHC-I promotes immune escape.** Tumorigenesis produces abnormal antigens on the tumor cell surface that can activate APCs (mainly DCs), and presentation of these tumor antigens to T cells activates the immune system. However, tumors often downregulate or alters the structure of MHC-I, thereby impairing antigen presentation and promotes immune escape (32). The equivalent molecule in humans, HLA-I, is often downregulated in TNBC patients resistant to ICB therapy (45). This could be due to defects in various HLA-I encoding genes and the invariant β2-microglobulin (β2m) gene (46). Transcription factors such as NF-κB and NLRC5 and epigenetic pathways are also critical for the regulation of HLA-I expression; the ablations of which will significantly affect antigen presentation (47). A clear example is interferon (IFN) which can induce the expression of HLA class I heavy chains (β2m, TAP1, TAP2 or Tapasin), and the impairment of which also lead to downregulation of HLA-I (48). In addition, genetic defects in any of the proteins involved in the MHC-I processing machinery, such as downregulation of the TAP transporters, will affect the processing and presentation of peptide antigens.

**Failure of APC recruitment and activation.** APC recruitment and activation are essential steps in the immune activation cascade. Reduction of chemokines that recruit APCs and downregulation of danger signals that activate them are all mechanisms that promote immune escape (49). Chemoattractants such as CCL5 and XCL1 that induce the accumulation of DCs are mainly produced by NK cells, while tumor-derived prostaglandin E2 (PGE2) can interfere with the expression of chemokines and their receptors in NK cell and DCs, respectively, and promote immune escape (50). Tumors can also hide danger signals to avoid APC phagocytosis by upregulating “don’t eat me” signals such as CD47, CD24, etc. (51, 52), and downregulate “eat me” signals including CRT, HMGB1, etc. (53). In TNBC, glycosylation of B7-H4 stabilizes and prevents the degradation of this protein, which inhibits eIF2α phosphorylation, leading to reduced surface expression of CRT and allowing the tumor to evade immune destruction (54).

**Dysfunction of APC maturation and immune initiation.** In addition to antigen presentation, mature DCs are required to provide co-stimulatory signals such as CD80, CD86, and CD40, that will activate T cells fully. Thus, factors such as type I IFN (IFN-I) is essential in promoting DC maturation and initiating adaptive immunity. Immature DCs are not only deficient in co-stimulatory molecules but also upregulate co-inhibitory receptors that can block T cell activation. Dendritic cells have been shown to express high levels of PD-L1, and downregulate CD80 expression, which prevents activation of T cells *via* CD28. This is thought to be one of the pathways that contribute to poor efficacy of ICB treatments.

#### 2.2.2 Tumor mutation load and epigenetic modification

Tumors can present a variety of neoantigens on their cell surface. Some of these are also present on healthy cells (tumor-associated antigens; TAAs), while others are unique to cancer cells (tumor-specific antigens; TSAs) (55). TSAs can arise from non-synonymous mutations, gene fusions, alternative splicing, and DNA damage responses in tumor cells. Variations in DNA copy number and genome instability may lead to gain or loss of neoantigens, thus affecting the immunogenic status of tumors. TSAs can stimulate T cell-mediated adaptive immunity; their abundance defines the tumor mutational burden (TMB), and patients with high TMB are generally more responsive to ICB treatment (56). Notably, PD-L1 expression is not significantly correlated with TMB in most cancer subtypes, suggesting that these two factors may contribute to distinct mechanisms of ICB resistance. Furthermore, interactions between the immune system and tumor cells exert selective pressure on cancer cells through the process of immunoediting and changes the trajectory of tumor development (57–59). Tumors with few TILs showed more evidence of past immunoediting events, such as reduced neoantigen abundance and heterogeneity, than those with more TILs or TMB (60); reflecting the ability of immunoediting to turn the tumor microenvironment from “hot” to “cold”.

In addition to mutations at the gene level, another immune escape mechanism occurs at the epigenetic level, including DNA methylation, RNA interference, histone modification, etc. Inhibition of immune gene expression through epigenetic modifications can negatively impact tumor immunity. For example, DNA methylation can lead to the silencing of immune genes (61), and loss of methylation may explain the paradoxical observation of low antitumor immune activities in tumors with high chromosomal copy number changes (62, 63), a possible mechanism by which highly mutated tumors resist immunity. In another contrary example, the cell cycle inhibitor CDK4/6 reduce the immunosuppressive Treg population by inducing hypomethylation of Treg genes through the Rb-E2F axis, thereby increasing tumor immunogenicity (64, 65).

### 2.3 Abnormal signaling pathway

**Figure 3:**
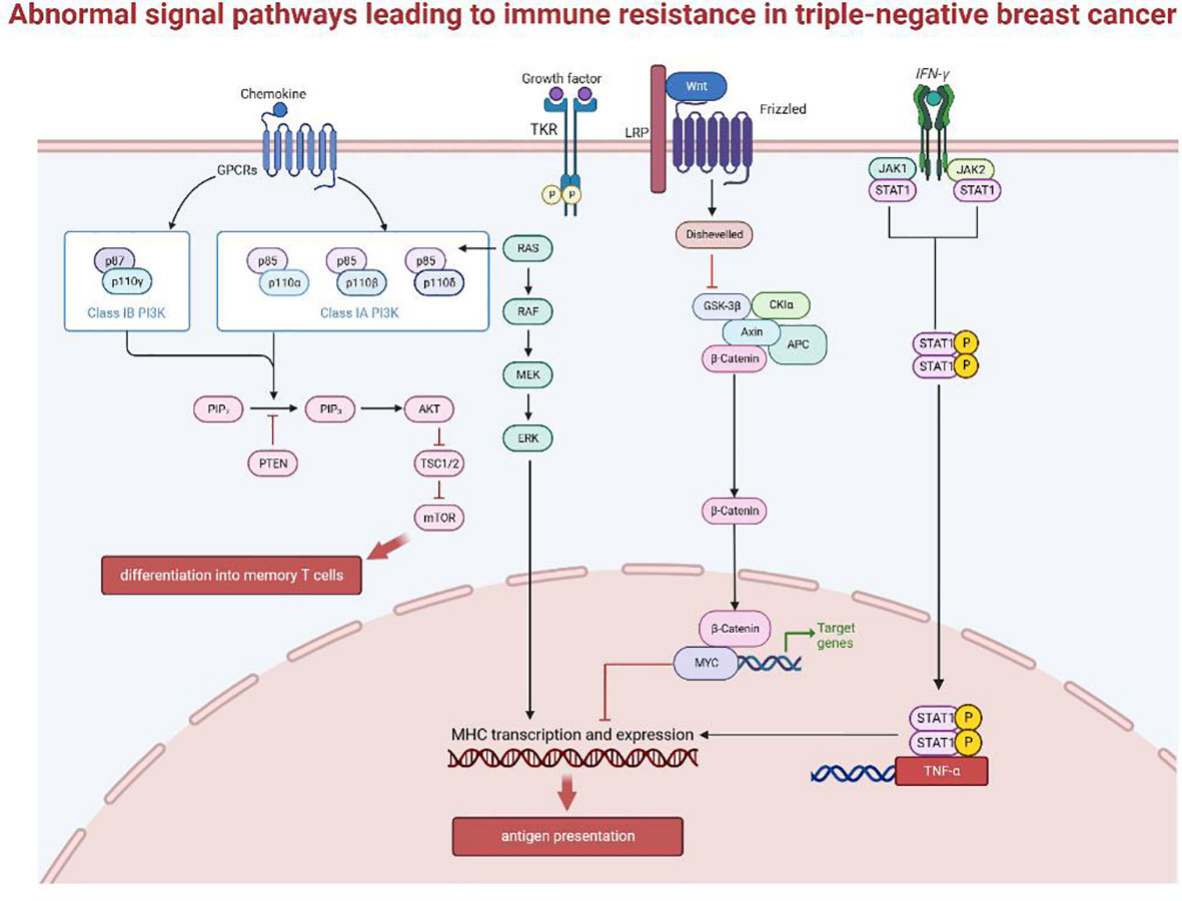
Abnormal signal pathways leading to immune resistance in triple-negative breast cancer.

Earlier we introduced how TNBC evades immune surveillance through external regulations and internal adaptations. Based on the above molecular mechanisms, the immunosuppressive microenvironment in tumors contributes to changes in immunogenicity, antigen presentation and the intensity of the inflammatory response, thus enabling cancer cells to escape immune surveillance or inhibit the recruitment and infiltration of lymphocytes. Signaling pathways that have been proven to be involved in immune escape are shown in **Figure 3**, including:

#### 2.3.1 The mitogen-activated protein kinase pathway

Abnormal activation of the MAPK pathway is intrinsically linked to tumor occurrence and development, and drug resistance mechanisms in various cancers. Data from the TCGA database showed that low TIL in the basal-like breast cancer subtype is correlated with activating changes in the Ras/MAPK pathway (66), including amplifications of KRAS, BRAF and RAF1, and truncation of NF1. At the same time, MEK expression can predict recurrence-free survival (RFS) and overall survival (OS), to a certain extent, for TNBC after neoadjuvant therapy (67). MEK activity appears to correlate negatively with expression of the antigen-presenting molecules, MHC-I and -II. The current accepted explanation for this is that the Ras/MAPK pathway can inhibit the inflammatory response mediated by IFNγ, thereby affecting the IFNγ-mediated expression of MHC-I, MHC-II, and PD-L1 and suppresses antigen presentation (68).

#### 2.3.2 WNT/β-catenin signaling pathway

The WNT/β-catenin signaling pathway is immunomodulatory in multiple cancers, including breast cancer. Tumor-intrinsic WNT/β-catenin signaling is aberrantly activated in non-inflamed tumors and prevents T cell infiltration into the tumor (69). WNT signaling may suppress immune activation through several mechanisms: (1) in TNBC, WNT expression in cancer stem cells increase PD-L1 expression, which can reciprocally regulate WNT at the transcriptional level. Together they drive the immunosuppressive microenvironment of the tumor and predict poor prognosis and immune resistance for TNBC (70). (2) β-catenin accumulation following WNT activation initiates transcription of downstream target genes, such as MYC, which has been associated with poor immune cell infiltration when expressed at high levels (71–73). MYC is highly expressed in TNBC and is negatively correlated with the expression of important genes expressed by MHC-I (BM2 (74), NLRC5 (75)), thus impairing antigen presentation. MYC can also epigenetically repress STING, a gene associated with autonomous immune responses, resulting in reduced chemokine production (including CCL5, CXCL10, and CXCL11) and TIL recruitment, ultimately impairing T cell-mediated immune surveillance (71). (3) Finally, the WNT pathway also regulates DC-mediated innate immunity (76, 77). β-catenin can stimulate the transcriptional repressor ATF3, which inhibits the transcription of CCL4 (69). Lack of CCL4 impairs activation of CD103+ DCs (78), and disrupts the activation and infiltration of CD8+ T cells (79), thus reducing the effectiveness of ICB treatments.

#### 2.3.3 IFN-γ signaling pathway

The main signaling pathway activated by IFN is the JAK-STAT pathway (80–82), involving phosphorylation and dimerization of the Janus kinases JAK1 and JAK2 up on IFN receptor binding, followed by transcriptional activation of STAT1 (signal transducer and activator of transcription1) (83, 84). IFN-γ has two opposing effects on anti-tumor immunity through the canonical JAK-STAT pathway (78). T cells are the main producers of IFN-γ, which in turn activates downstream target genes such as TNF-α, iNOS, COX-2 and IL-1β, leading to enhanced expression of MHC, antigen presentation, recruitment of immune cells, and activation of inflammatory pathways (81). However, long-term IFN-γ exposure exerts selective pressure on tumors. Enrichment of genes such as IFN-γ receptor, JAK2, and interferon regulatory factor 1 were observed in patients who are unresponsive to anti-CTLA-4 therapy (84). Therefore, cancer cells can downregulate or mutate molecules involved in the IFN-γ signaling cascade through immunoediting, ultimately leading to immune evasion (85, 86). Cancer cells can also increase the transcription and expression of PD-L1 as a negative feedback mechanism (87). The role of type I IFN in antitumor immunity does not completely overlap with that of IFN-γ. Their mechanisms of function, the nature of the responding cell population, and the type of response induced are not very clear (88), and further studies are required.

#### 2.3.4 PTEN-PI3K/Akt signaling pathway

Lack of PTEN can contribute to ICB treatment resistance by affecting the recruitment and function of T cells. PTEN loss increases the expression of immunosuppressive factors, such as CCL2 and VEGF. Anti-VEGF antibodies has been shown to enhance the infiltration and activity of T cells in the tumor (89, 90). Thus, PTEN loss likely decrease T cell infiltration *via* increased VEGF expression. PTEN loss also blocks T cells cytotoxicity by inhibiting autophagy (91), since restoring the expression of autophagy-related genes, such as ATG16L and LC3 (92, 93), in PTEN-silenced cancer cells can increase cancer cell resistance to autologous TIL-induced apoptosis. These two pathways inhibit T cell recruitment and function, respectively, leading to immune resistance (94).

PTEN also suppresses the PI3K/Akt pathway by dephosphorylating PIP3 (95). Existing studies on the targeting of Akt in tumor therapy found that the extend of Akt activation can regulate CD8+ T cell differentiation, with sustained Akt signaling producing short-lived effector cells (SLECs) and reduced Akt levels directing differentiation of memory precursor effector cells (MPECs). Thus, Akt activation levels direct the fate of effector T cells to generate a heterogeneous population (96). Mechanistically, Akt regulates the transcriptional program triggered by T cell receptor (TCR) signaling and interleukin-2 (IL-2), to drive the expression of key adhesion and cytolytic molecules that differentiate effector versus memory T cells (97). Akt-targeted therapy improves the persistence of T cells (98), and shows great potential in TIL adoptive cell therapy (ACT), and may be a promising strategy to overcome drug resistance in breast cancer.

## 3 Discussion

Understanding the interactions between the tumor and the host immune system can shed lights on the processes and mechanisms of immunogenic resistance, and identify potential targets for intervention. Mechanisms that contribute to drug resistance in TNBC, including primary and adaptive drug resistance, can be summed up in the following three points: (i) weaken immunogenicity of the tumor; (ii) diminished antigen presentation by MHC; (iii) reduced recruitment and infiltration of immune cells. Analysis of the human immune landscape shows that the occurrence of TNBC immune resistance is the result of the combined interaction of multiple mechanisms within the tumor ecosystem (26).

Research on the mechanism of drug resistance in TNBC is still lacking in several aspects. First, there is no unified immune phenotyping for TNBC, which leads to a lack of precision in the research on the mechanism of immune resistance for this disease. Due to the highly heterogenous and complex nature of TNBC, PD-L1 expression or TIL abundance alone cannot accurately predict the effect of immunotherapy (99), and the diagnosis of immune resistance still lacks accurate predictive biomarkers (100). Second, the contribution of external factors on TNBC immune resistance is not understood, and the complex molecular interactions within the TME require more studies. Finally, strategies to reverse immune drug resistance is currently still in the preclinical stage and actual clinical effects are unclear. Therefore, based on the concept of precision therapy, we propose the following prospects for the above shortcomings:

(1) Break away from the simplistic classifications of tumors into “cold” or “hot” immunophenotypes. Galon et al. proposed an “immune environment” that describes the tumor immune state based on the combination of all immune variables related to the nature, density, orientation and distribution of immune cells in the tumor (97), which challenges the tradition “cold” and “hot” tumor concept. Thus, we should strive to define new, standardized tumor immunophenotypes that will be helpful to guide immunotherapy decisions. Attempts have already been made for colorectal cancer (CRC) where Camus et al. described three main immune features, immune-hot, altered and cold (101), which led to a new classification standard based on the balance of immune escape and immune regulation. Subsequently, Galon et al. proposed a fourth classification in which the “altered” phenotype is divided into two subtypes, “excluded” and “immunosuppressed” (101). The difference between these two subtypes of tumors is that the T cells of the former are distributed around the tumor and cannot infiltrate the tumor, but retain the ability to activate and initiate immune functions, while the latter tumor type show low T cell infiltration and have an TME that limits T cell recruitment and proliferation. These new immunophenotyping categories have been recognized as better predictors of patient response to ICB (102). In addition, Shao et al. analyzed the clinical, genomic and transcriptomic data of a Chinese TNBC cohort of 465 cases and found four transcriptome-based subtypes, luminal androgen receptor (LAR), immunomodulatory (IM), basal-like immune-suppressed (BLIS), and mesenchymal-like (MES) (103). Among them, the IM subtype had more lymphocyte infiltration around the tumor cells and showed a high sensitivity to immunotherapy compared with other subtypes. All in all, the immunophenotyping of TNBC has not yet formed a globally unified standard and consensus, and more tumor immunotypes are shown in **Table 1**. Developing different therapeutic strategies for tumors of specific immune subtypes will help achieve precision treatment in the clinic.

**Table 1:** Summary of the different systems of classifying the immunotype of tumors.

Initial proposed time	Cancer type	Tumor immunotypes	Basis of classification
/	All cancers	immune-hot and immune-cold	response of cancer patients to ICB therapy
2009 Camu, M	CRC	immune-hot, altered and cold	according to the balance between tumor escape and immune coordination
2014 Galon, J.	CRC	TNM-Immunes: 10-14	Immunoscore: CT+IM teo lymphocyte populations: in the core of the tumor (CT) and in the invasive margin of tumors (IM)
2017 Chen, D.S	All cancers	inflamed tumor, immune-desert tumor	Cancer-immune set point: represents the threshold that must be surpassed for a person with cancer to respond to immunotherapy
2019 Galon, J.	All cancers	hot, altered-excluded, altered-immunosuppressed and cold	based on the cytotoxic T cell landscape within a tumor
2019 Jiang, Y.Z	Triple-Negative Breast Cancer (TNBC)	luminal androgen receptor (LAR), immunomodulatory (IM), basal-like immune-suppressed (BLIS), mesenchymal-like (MES)	the clinical, genomic and transcriptomic data of a Chinese TNBC cohort of 465 cases
2020 Desboi, M.	Ovarian Cancer	Immunoreactive (IMR), Mesenchymal (MES), proliferative (PRO) differentiated (DIF)	Digital pathology and transcriptome analysis of a large ovarian tumor cohort
2020 Zhang, B	Gastric Cancer	imunne-excluded phenotype, immune-inflamed phenotype, immune-dessert phenotype	three distinct m6A methylation modification pattterns

(2) Improve understanding of external mechanisms driving tumor drug resistance. The tumor microenvironment is a complex ecosystem composed of a variety of cellular and non-cellular components. Crosstalk between tumor cells, immune cells and the tumor stroma co-develops the immunosuppressive microenvironment, creating conditions for immune evasion (104). For example, cancer-associated fibroblasts (CAFs) can secrete a series of cytokines to suppress the immune response, VEGF to regulate the tumor vascular network, TGF-β to inhibit DC maturation and promote Treg differentiation etc., and hinder the infiltration of drugs and immune cells through the extracellular matrix (105, 106). The acidic environment of the TME also inhibits the activity of immune cells, and metabolites such as lactic acid produced in a hypoxic environment can limit the function of effector T cells and promote immune regulatory functions of Tregs (107). Thus, non-immune cellular components of the tumor environment may also participate in the formation of a highly immunosuppressive microenvironment, and the dynamic balance between these factors determines the immune response and efficacy of antitumor therapy, and are promising research directions.

(3) Translating drug resistance targets to the clinical. The following strategies are proposed for the drug resistance mechanisms summarized above: (i) improve the immunogenicity of tumors; (ii) increase antigen presentation by MHC; (iii) regulate the recruitment and infiltration of immune effector cells. Considering the key role of T cells against cancer, these strategies all aim to increase the abundance of TILs within the tumor, so as to recover antitumor immune activities. Combination immunotherapies based on the above ideas have been shown to be superior to monotherapy in several randomized clinical trials (108), achieving significant clinical benefits, and demonstrating the great potential and broad prospects of combination immunotherapy. However, not all drug-resistant targets can show their effects beyond preclinical models, and we speculate that their limitations lie in the responsiveness of cancer patients to immunotherapy and is co-determined by the inherent immunogenicity of the tumor and the reactivity of the individual immune system. Thus, differences in tumor immunogenicity arising from heterogeneity between TNBC patients, and even at different sites within the same tumor, and individual differences in reactivity arising from heterogeneity of the TME collectively lead to an overestimation of the efficacy of strategies to reverse resistance. Therefore, strategies for reversing drug resistance urgently require more comprehensive experimental designs and clinical trials to narrow the gap between preclinical results and clinical applications and pave the way for clinical translation.

## 4 Conclusion

With in-depth studies of the process and molecular mechanism of TNBC immune resistance, we need methods to assess multiple immune variables to find predictive biomarkers that will identify appropriate immune characteristic subgroups. This will guide immune stratification, treatment plan selection, and improve the predictability and efficacy of TNBC immunotherapies in a new era of truly personalized medicine.

## Author contributions

YZ, SL drafted the manuscript and QZ critically revised the manuscript. YZ, SL and HT designed the figures and tables. QZ and XM conceived and critically revised the manuscript and tables. All authors contributed to the article and approved the submitted version.
